# Ultra-Fast Removal of CBB from Wastewater by Imidazolium Ionic Liquids-Modified Nano-Silica

**DOI:** 10.3390/molecules30010024

**Published:** 2024-12-25

**Authors:** Mengyue Zhang, Fan Yang, Nan Wang, Jifu Du, Juntao Yan, Ya Sun, Manman Zhang, Long Zhao

**Affiliations:** 1 School of Chemical and Environmental Engineering, Wuhan Polytechnic University, Wuhan 430040, China; 2 State Key Laboratory of Advanced Electromagnetic Engineering and Technology, School of Electrical and Electronic Engineering, Huazhong University of Science and Technology, Wuhan 430074, China; 3 School of Nuclear Technology and Chemistry & Biology, Hubei University of Science and Technology, Xianning 437100, China

**Keywords:** nano-silica, ionic liquids, dye, Coomassie brilliant blue

## Abstract

The efficient removal of dyes is of significant importance for environmental purification and human health. In this study, a novel material (Si-MPTS-IL) has been synthesized by the immobilization of imidazole ionic liquids (ILs) onto nano-silica using the radiation grafting technique. The adsorption performance of Si-MPTS-IL for Coomassie Brilliant Blue (CBB) removal is studied by a series of static adsorption experiments. It is found that Si-MPTS-IL has ultra-fast adsorption kinetics, reaching equilibrium within 2 min. The adsorption process for CBB conforms to the Langmuir model. In addition, Si-MPTS-IL exhibits a negligible impact on the adsorption efficiency of CBB with the increase in salt concentration. After six cycles of adsorption–desorption, the adsorption efficiency of Si-MPTS-IL remained above 80%, indicating excellent regenerative properties and a promising candidate for the treatment of wastewater containing CBB. A study of the mechanism indicates that the CBB capture by Si-MPTS-IL can be attributed to the synergistic effects of electrostatic interactions and pore filling.

## 1. Introduction

Organic dyes represent a major source of water pollution, primarily resulting from effluent discharge by various industries, including textiles, plastics, printing, photography, paper-pulp, paints and leather processing [1,2,3,4]. Particularly, Coomassie Brilliant Blue (CBB) is an anionic synthetic dye containing triphenylmethane, commonly used for protein staining and evaluation in clinical and biochemical laboratories. It is considered a toxic and persistent organic pollutant due to its non-degradability and environmental pollution [5,6]. Therefore, the removal of CBB from wastewater is critical to mitigating its detrimental effects on ecosystems and human health [7].

Several methods have been developed and applied to remove the anionic dye from water and wastewater, such as membrane separation [8,9], adsorption [10,11,12], chemical oxidation [13], photodegradation [14,15] and bioremoval [16,17]. Chemical oxidation can completely mineralize the organic pollutants and is suitable for treating high-concentration wastewater. However, it requires a significant consumption of chemicals, leading to high treatment costs and the potential generation of secondary pollutants. Bioremoval is environment-friendly and sustainable, making it ideal for handling low-concentration pollutants. Nevertheless, it is characterized by slow degradation rates and requires specific conditions (e.g., pH, temperature) to maintain microbial activity. Among them, adsorption has been proven to be an efficient approach in terms of both technology and economy because of its low process cost, ease of operation and high efficiency [13]. Currently, activated carbon, porous organic materials, metal–organic frameworks, etc. are all applied in the dye-capture process [18,19,20,21]. Moreover, the adsorbent materials can often be recycled and reused. Activated carbon, a commonly used adsorbent for CBB removal, achieves an adsorption capacity of 150–200 mg/g with a removal efficiency of approximately 85%. However, its regeneration process is energy-intensive. There are some challenges in terms of synthesis costs and practical applications. From this perspective, adsorbents with high adsorption capacity and cost-effectiveness are urgently required.

Nano-silica exhibits excellent pore size and specific surface area [22,23]. However, its surface is deficient in functional groups, necessitating modification. Researchers have identified that functionalized silica demonstrates exceptional adsorption capacity for dyes [24]. Therefore, selecting functional groups that exert strong interactions with CBB is critical for the development of adsorbents with superior adsorption performance. In recent years, ionic liquids (ILs) have been widely used for the adsorption and separation of dyes in wastewater due to the strong electrostatic attraction [25]. However, there are disadvantages to using ILs as extractants, including high consumption rates and difficulties in recovery [26]. Thus, the efficient immobilization of ILs onto nano-silica can fully leverage the primary advantages, resulting in the development of novel adsorbent materials that meet the desired specifications.

In this work, a novel nano-adsorbent material (Si-MPTS-IL) was prepared by immobilizing imidazole ILs onto a nano-silica substrate using electron beam irradiation technology. The Si-MPTS-IL was characterized by FTIR, SEM and TGA. The adsorption properties of the material towards CBB were investigated through a series of static adsorption experiments.

## 2. Results and Discussion

### 2.1. Preparation and Characterization of Si-MPTS-IL

**Figure 1:**
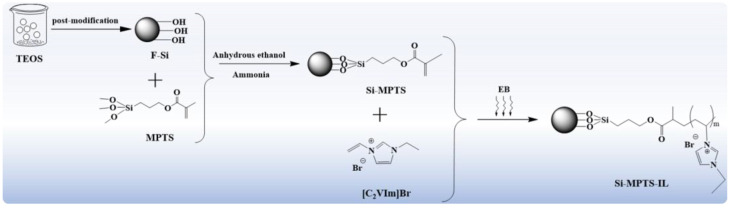
Synthesis route of Si-MPTS-IL.

**Figure 2:**
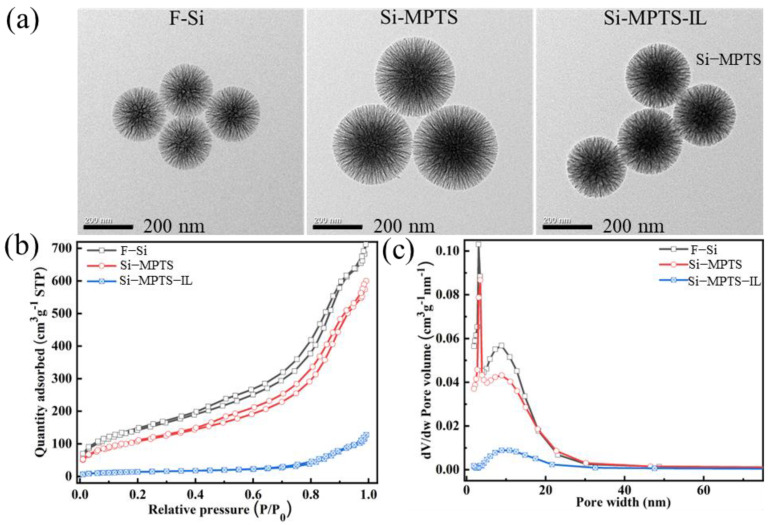
TEM (**a**), N_2_ adsorption–desorption isotherms (**b**) and pore width distribution (**c**) of F-Si, Si-MPTS and Si-MPTS-IL.

As presented in Figure 1, the nano-silica particles (F-Si) were prepared using pure tetraethyl orthosilicate (TEOS). Then, the silanized nano-silica (Si-MPTS) was prepared by a nucleophilic substitution reaction between F-Si and 3-(isobutenyloxy) propyl trimethox-ysilane (MPTS). Finally, the radiation technology was used to initiate the vinyl polymerization of Si-MPTS and 1-vinyl-3-ethylimidazolium bromide ([C_2_VIm]Br), resulting in the imidazolium ionic liquids-modified nano-silica (Si-MPTS-IL). The conversion rate of ILs on Si-MPTS-IL was 37%. The structure and morphology of Si-MPTS-IL were characterized by TEM, FT-IR and TGA. The Brunauer–Emmett–Teller (BET) surface area was calculated using N_2_ adsorption isotherms. The morphology of F-Si, Si-MPTS and Si-MPTS-IL are shown in Figure 2a. As can be observed, F-Si has a fibrous structure and obvious pores with a size of about 300–400 nm, which is conducive to the efficient removal of CBB. The morphologies of Si-MPTS and Si-MPTS-IL remains unchanged, indicating that these processes would not result in structural damage to nano-SiO_2_.

In Figure 2b,c, the specific surface area and pore size distribution of the adsorbent were investigated through N₂ adsorption–desorption experiments. The N₂ desorption curves for Si-MPTS-IL conform to type IV isotherms, indicating mesoporous materials with the pore size distribution ranging from 5 to 25 nm, providing more active adsorption sites and effective transport pathways for the adsorption of CBB. Following alkylation, the specific surface area decreased from 535 m²/g (F-Si) to 399 m²/g (Si-MPTS), while the total pore volume decreased from 1.101 m³/g to 0.9278 m³/g. The reduction in specific surface area and pore volume after alkylation and grafting suggests that these reactions occurred within the internal pores and on the surface of the nano-silica.

The FT-IR spectra of F-Si, Si-MPTS and Si-MPTS-IL are provided in Figure S1a. The distinctive peak at 3446 cm^−1^ belongs to the OH group; 1095 cm^−1^ and 806 cm^−1^ were the characteristic peaks of Si-O-Si, indicating the successful synthesis of silica. The peaks at 1720 cm^−1^ represent the C=O groups of MPTS, proving that the silica was silanized successfully [27]. After grafting [C_2_VIm]Br, new peaks at 1571 cm^−1^, 1554 cm^−1^ and 655 cm^−1^ appeared in the infrared spectrum, corresponding to C=C, C=N and C-H on the imidazole ring, respectively, which proved the successful grafting of imidazolium IL [28]. The thermogravimetric analysis shown in Figure S1b also indicates that Si-MPTS-IL was synthesized successfully.

### 2.2. Batch Adsorption Experiments

#### 2.2.1. pH Effect

**Figure 3:**
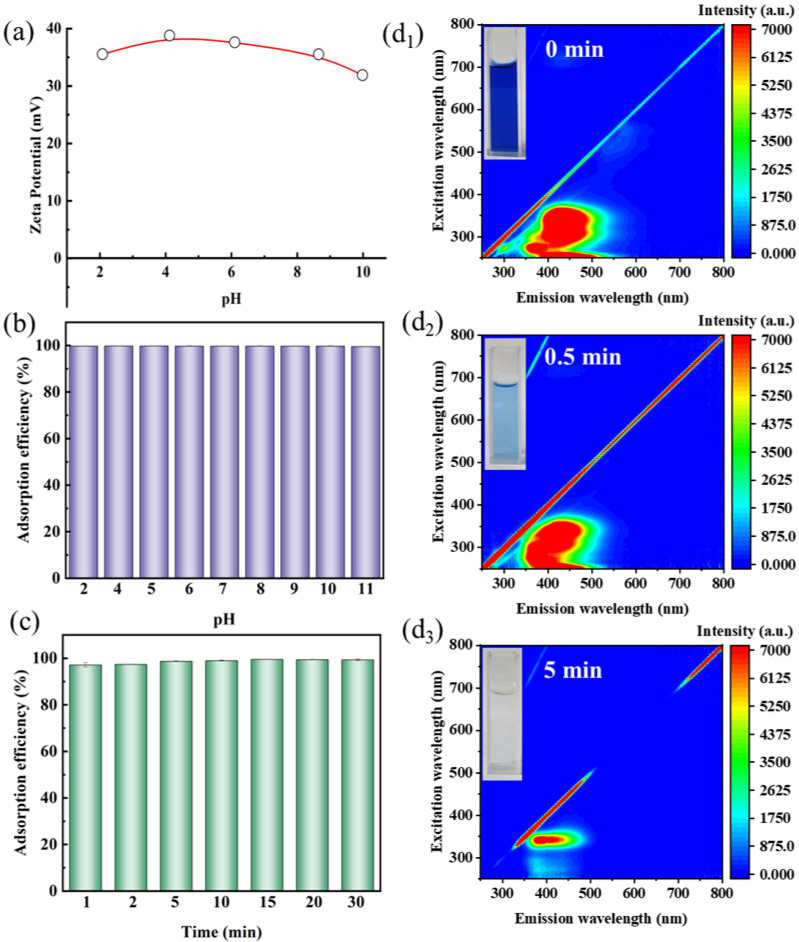
Zeta potential of Si-MPTS-IL (**a**); Effect of pH (**b**) and contact time (**c**) on CBB uptake; (**d**) 3D fluorescence spectrogram during adsorption on Si-MPTS-IL.

The pH of the solution is a critical factor in the adsorption process, as it impacts the surface charge of both the adsorbent and the adsorbate molecules. The Zeta potential of Si-MPTS-IL at varying pH values is illustrated in Figure 3a. Notably, the Zeta potential of Si-MPTS-IL consistently exceeds +30 mV over the whole pH range. Generally, a high Zeta potential is associated with an enhanced electrostatic attraction for the anionic species (CBB). The effect of solution pH on the CBB capture is depicted in Figure 3b. It is found that the adsorption efficiency of CBB by Si-MPTS-IL remains stable within the pH range of 2 to 11, likely due to the electrostatic interactions between the positively charged sites on the adsorbent and the negatively charged CBB molecules. The sustained high adsorption efficiency of Si-MPTS-IL over a broad pH range underscores its significant potential for practical applications.

#### 2.2.2. Adsorption Kinetics Studies

The influence of contact time on the adsorption efficiency of CBB on Si-MPTS-IL was investigated in Figure 3c. Initially, the adsorption rate of CBB was rapid, reaching equilibrium within 2 min. In the 3D fluorescence analysis (Figure 3d), a significant reduction in the peak intensities of CBB is observed with increasing adsorption time, indicating effective capture of Si-MPTS-IL towards CBB. This phenomenon can be primarily attributed to the available active sites on Si-MPTS-IL, resulting in a high adsorption rate in a short time. In comparison with other materials (Table S1), Si-MPTS-IL exhibited rapid adsorption kinetics, highlighting its high efficiency for potential practical applications.

To elucidate the kinetic process of CBB uptake on Si-MPTS-IL, the experimental adsorption data of CBB were fitted with the pseudo-first-order kinetic (PFO) (Equation (S1)) and the pseudo-second-order kinetic model (PSO) (Equation (S2)). The fitting results are illustrated in Figure S2 and Table S2. Notably, the fitting coefficient of PSO is higher than the PFO model, indicating that the adsorption of CBB by Si-MPTS-IL is more accurately described by the PSO model. The increase in adsorption rate as the adsorbate concentration rises supports this model, indicating that the adsorption process is predominantly governed by the concentration of available active sites rather than external factors such as mass transfer.

#### 2.2.3. Adsorption Isotherms and Thermodynamics

**Figure 4:**
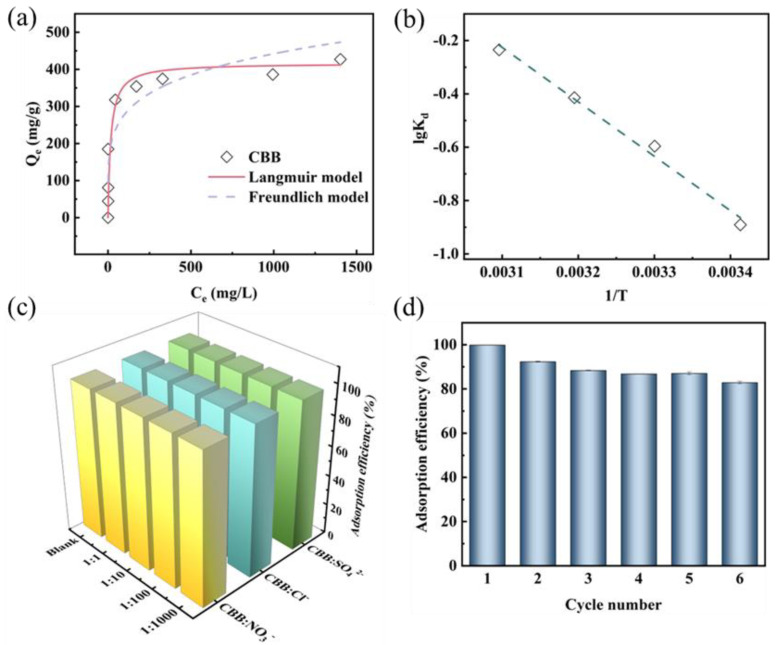
Effect of initial concentration (**a**), temperature (**b**), salt concentration (**c**) and cycle number (**d**) on the adsorption of CBB by Si-MPTS-IL.

The relationship between the adsorption capacity of Si-MPTS-IL and CBB concentration is explored by the adsorption isotherm. In Figure 4a, the adsorption capacity of Si-MPTS-IL for CBB increases initially with increasing concentrations and eventually approaches equilibrium. This is because a higher number of adsorption sites are available at the beginning. However, with the continuing rise in CBB concentration, the adsorption sites gradually become occupied, leading to adsorption saturation. Additionally, the large specific surface area and porous structure of the adsorbent are also favorable for CBB uptake by Si-MPTS-IL.

To assess the adsorption capacity, the Langmuir (Equation (S3)) and Freundlich (Equation (S4)) models are selected to fit the isothermal data. The fitting plots and parameters are presented in Figure 4a and Table S3. Upon comparing the R^2^ values and adsorption capacity, the Langmuir model (R^2^ > 0.99) proved suitable for describing the CBB removal, indicating the predominance of monolayer adsorption in the process. The maximum adsorption capacity of Si-MPTS-IL for CBB is 414.44 mg/g, exceeding the values reported for other adsorbents in prior studies (Table S1) [29,30,31,32,33]. Additionally, the effect of temperature on the adsorption performance is explored in Figure 4b and Table S4. The study reveals that the adsorption process of CBB onto Si-MPTS-IL is endothermic. In summary, Si-MPTS-IL is regarded as having excellent adsorption performance, making it a promising candidate for the treatment of wastewater containing CBB.

#### 2.2.4. Effect of Salt Concentration and Coexisting Dyes

Industrial waste generated from the textile industry is typically complex, containing various types of inorganic salts. Therefore, to investigate the practical application performance of Si-MPTS-IL, the effects of representative salts (NaNO₃, NaCl and Na₂SO₄) on the adsorption performance for CBB are explored in Figure 4c. As the salt concentration increased, there was negligible impact on the adsorption efficiency of CBB by Si-MPTS-IL. Notably, even at a salt concentration 1000 times greater than that of CBB, Si-MPTS-IL maintained superior selectivity for CBB.

The specificity of the Si-MPTS-IL material towards CBB is an important aspect of our study. As shown in Figure S3, Si-MPTS-IL exhibits a relatively high affinity for CBB, which is primarily due to the ionic interactions between the ethylimidazolium groups and the anionic dye molecules. However, the material may also exhibit some affinity for other similar contaminants, particularly those with similar charge characteristics or molecular structures, due to the non-specific adsorption sites on the surface of Si-MPTS-IL.

#### 2.2.5. Recycling Experiment

The regenerability of the adsorbent is crucial for enhancing industrial efficiency and reducing costs. The reusability of Si-MPTS-IL is investigated through six cycles of adsorption–desorption using 0.5 mol/L HNO_3_ as eluent in Figure 4d. It is clearly observed that although the adsorption efficiency of Si-MPTS-IL for CBB shows a slight decrease with increasing cycles, it remains above 80% after six cycles of adsorption–desorption, indicating that Si-MPTS-IL possesses excellent reusability.

### 2.3. Adsorption Mechanism

**Figure 5:**
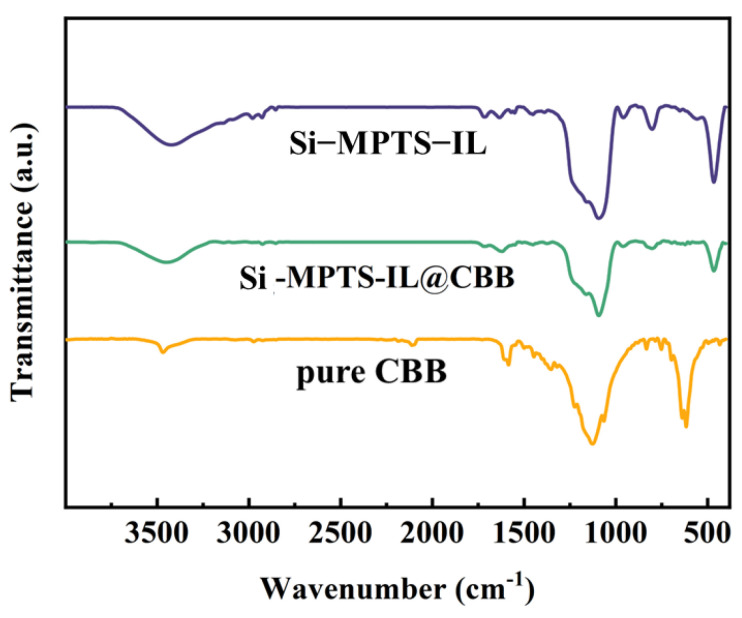
FT-IR spectra of Si-MPTS-IL before and after adsorption.

FTIR spectroscopy analysis helps to analyze the surface changes of adsorbent materials after adsorbing CBB and helps propose the interaction mechanism. In Figure 5, the peak at 1342 cm^−1^ is attributed to the vibration of C-N. The peaks observed at 1240 cm^−1^, 1168 cm^−1^ and 1072 cm^−1^ correspond to the oxygen stretching of the sulfonic acid group (-SO_3_) in the FTIR spectrum of CBB. After Si-MPTS-IL adsorbed CBB, characteristic peaks of CBB appeared at 1344 cm^−1^, 1236 cm^−1^ and 1170 cm^−1^, which are related to the absorption band of the sulfonic acid (-SO_3_), indicating that CBB is anchored to the adsorbent material [34]. The physical and chemical interactions between the adsorbent and the adsorbate can jointly promote the CBB removal; these interactions include electrostatic interactions, ion exchange and pore filling [35]. Combining the specific surface area, pore size distribution (Figure 2) and the pH experimental results (Figure 3b), the CBB capture by Si-MPTS-IL can be attributed to the synergistic effects of electrostatic interactions and the pore filling.

Additionally, the DFT calculations were conducted to demonstrate that Si-MPTS-IL can effectively adsorb CBB. To optimize the geometry of the potential Si-MPTS-IL@CBB in an aqueous solution, the geometry optimization feature in the Dmol 3 module of Materials Studio 7.0 was utilized. The structures of the material before and after CBB adsorption were optimized using the GGA/BP functional without imposing any symmetry constraints. During the optimization process, all atoms were fully relaxed. The adsorption energy (ΔE_ads_) was determined using the following Equation (1):

ΔE_ads_ = E_adsorbent-CBB_ – (E_adsorbent_ + E_CBB_) (1)

The electrostatic interactions and optimized geometries between Si-MPTS-IL and CBB could be simplified as shown in Table 1. The negative ΔE indicated that the adsorption process benefited from the electrostatic interactions process.

## 3. Materials and Methods

### 3.1. Materials and Chemicals

1-vinyl-3-ethylimidazolium bromide ([C_2_VIm]Br, 99%) was supplied by Green Chemistry and Catalysis Center, Lanzhou Institute of Compounds, Chinese Academy of Sciences. Tetraethyl orthosilicate (TEOS) was purchased from National Non-ferrous Metals and Electronic Materials Analysis and Testing Center. Cetyltrimethylammonium bromide (CTAB, 99%) and 3-(isobutenyloxy)propyl trimethoxysilane (MPTS, 97%) were sourced from Aladdin Biochemical Technologies Inc. The Coomassie Brilliant Blue (CBB) was obtained from Shanghai McLean Biochemical Technology Co., Ltd. Table S5 shows the chemical structures and general characterization of CBB.

### 3.2. Synthesis of Imidazolium Ionic Liquids-Modified Nano-Silica

Firstly, Si-MPTS was prepared based on our previous work [36], and the synthesis route is illustrated in Figure 1. Secondly, 0.1g of Si-MPTS was weighed into a polyethylene bag for vacuum sealing, and then the 30 wt% [C_2_VIm]Br was injected under the condition of nitrogen flow deoxidation. The electron accelerator (WasiK, USA, Energy 1 MeV, 20 kGy/pass) was used for beam irradiation at 120 kGy. After irradiation, non-reactive monomers and homopolymers were washed for several times with deionized water and anhydrous ethanol. Finally, Si-MPTS-IL was obtained by centrifugation and dried at 50 °C (the specific reaction details are given in Supporting Information). The conversion rate of the functionalization process can be quantified using Equation (2):

(2) $$C\mathrm{onversion}\;rate\;\left(\%\right)=\frac{M_{g}-M_{0}}{M_{0}}$$

where *M*_0_ denotes the mass of Si-MPTS, while *M*_g_ signifies the mass of Si-MPTS-IL.

### 3.3. Characterization

Fourier transform infrared spectroscopy (FT-IR) was performed using a Bruker Tensor 27 in the wavenumber range of 4000−400 cm^−1^. The thermal stability was evaluated through TGA, utilizing a TA Instruments model 600 (New Castle, USA). The morphology of adsorbed materials was characterized bythe emission transmission electron microscopy (TEM, FEI Tecnai G2 F30, FEI Corporation, Hillsboro, OR, USA). The CBB solution before and after adsorption are subjected to a 3D fluorescence test (SmartFluo-Pro, Zolix Instruments Co., Ltd., Beijing, China) to compare the adsorption performance of Si-MPTS-IL. The potential of Si-MPTS-IL at different pH values was analyzed using a nano-sized potential analyzer (BeNano Zeta, Dandong Baiter Instrument Co., Ltd., Dandong, China). The concentration of CBB in the solution before and after adsorption was tested using a UV-2700 spectrophotometer (Shimadzu Corporation, Kyoto, Japan).

### 3.4. The Conditions of Batch Adsorption Experiments

The adsorption behavior of Si-MPTS-IL for CBB was investigated through batch experiments. Specifically, 10 mL of CBB solution was combined with 0.01 g of Si-MPTS-IL. The mixture was shaken at 160 rpm and 30 °C for a certain time. After adsorption, the mixture was collected and filtered through 220 nm polyethersulfone (PES) filter. The effect of pH, contact time, initial concentration, temperature, and salt concentration on the removal efficiency of CBB onto Si-MPTS-IL was investigated by a similar method. The adsorption capacity (q*_t_*) and adsorption efficiency (*E*) were determined using the equations below (Equations (2) and (3)):

(3) $$q_{t}=(C_{0}-C_{t})\times V/m$$

(4) $$E(\%)=(C_{0}-C_{t})\times 100/C_{0}$$

where C_0_ (mg/L) and C*_t_* (mg/L) are the initial concentration and equilibrium concentration of CBB, respectively. V (mL) and *m* (mg) are the volume of the solution and the mass of Si-MPTS-IL, respectively.

## 4. Conclusions

In conclusion, the novel IL-immobilized nano-silica (Si-MPTS-IL) was successfully prepared through radiation grafting technology. The adsorption results indicated that Si-MPTS-IL can efficiently remove CBB over a wide pH range. Si-MPTS-IL exhibited rapid adsorption kinetics for CBB, reaching equilibrium within 2 min. The maximum adsorption capacity for CBB was 414.41 mg/g, according to the Langmuir mode. Furthermore, the adsorption efficiency of Si-MPTS-IL for CBB was not affected at high salt concentrations. After six cycles of adsorption–desorption, the adsorption efficiency of Si-MPTS-IL remained above 80% of the original value, indicating excellent regenerative properties and making it a promising candidate for the treatment of wastewater containing CBB.

## Figures and Tables

**Table 1:** The electrostatic interactions between Si-MPTS-IL and CBB.

CBB	Si-MPTS-IL	Si-MPTS-IL@CBB	ΔE_ads_ (eV)
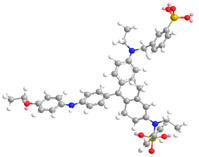	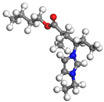	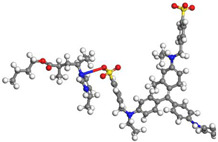	−4.5471

## Data Availability

The data used in this work are available on request from the corresponding authors.

## Supplementary Materials

The following supporting information can be downloaded at https://www.mdpi.com/article/10.3390/molecules30010024/s1: Figure S1: FTIR spectra (a) and TGA (b) of F-Si, Si-MPTS and Si-MPTS-IL; Figure S2: Fitted kinetic curves of Si-MPTS-IL for CBB; Figure S3: The adsorption performance of Si-MPTS-IL for different dyes; Table S1: Comparison of adsorption capacity and time of Si-MPTS-IL for CBB and other materials; Table S2: Kinetic parameters for CBB adsorption by Si-MPTS-IL; Table S3: Isotherm fitting parameters of Si-MPTS-IL for CBB; Table S4: Thermodynamic fitting parameters of adsorption of CBB by Si-MPTS-IL; Table S5: Chemical structure and general characterization of CBB.

## Abbreviations

[C_2_VIm]Br	1-vinyl-3-ethylimidazolium bromide
TEOS	Tetraethyl orthosilicate
CTAB	Cetyltrimethylammo-nium bromide
MPTS	3-(isobutenyloxy)propyl trimethoxysilane
CBB	Coomassie Brilliant Blue
