# Supplementary material for: Ultra-Fast Removal of CBB from Wastewater by Imidazolium Ionic Liquids-Modified Nano-Silica
Source: Molecules. 2024 Dec 25;30(1):24. doi: 10.3390/molecules30010024 (PMC11721558; doi:10.3390/molecules30010024)
Supplement: Supplementary file 1 [file molecules-30-00024-s001.zip › molecules-3356033-supplementary.pdf]

Supporting Information

Ultra-fast removal of CBB from wastewater by imidazolium ionic liquids modified nano-silica

Mengyue Zhang[1], Fan Yang[1], Nan Wang[2], Jifu Du[3], Juntao Yan[1], Ya Sun[1], Manman Zhang[1]*, Long Zhao[2]*

[1] School of Chemical and Environmental Engineering, Wuhan Polytechnic University, Wuhan 430040, China.

[2] State Key Laboratory of Advanced Electromagnetic Engineering and Technology, School of Electrical and Electronic Engineering, Huazhong University of Science and Technology, Wuhan 430074, China.

[3] School of Nuclear Technology and Chemistry & Biology, Hubei University of Science and Technology, Xianning 437100, China.

[*] Corresponding author at: School of Electrical and Electronic Engineering, Huazhong University of Science and Technology, Wuhan 430074, China (Long Zhao), E-mail address: zhaolong@hust.edu.cn; ryuuchou@hotmail.com.

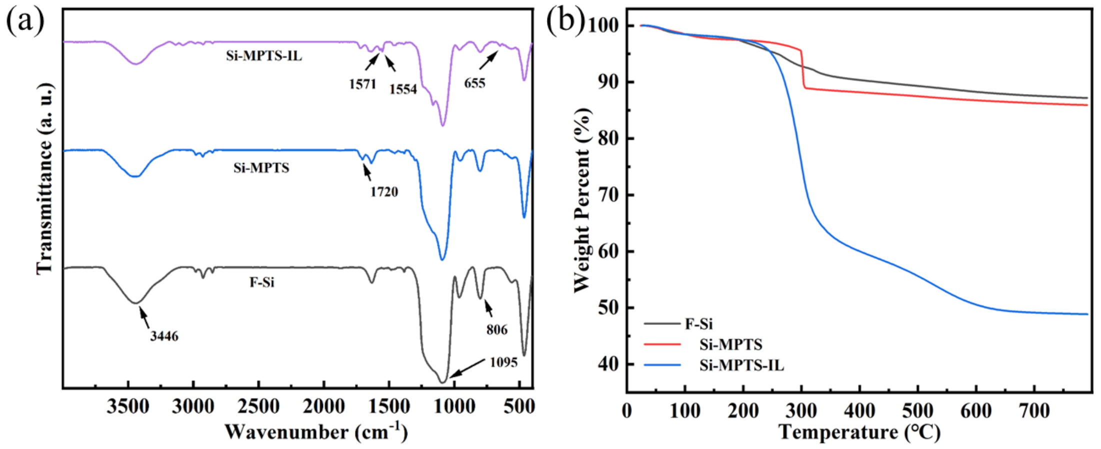

Figure S1. FTIR spectra (a) and TGA (b) of F-Si, Si-MPTS and Si-MPTS-IL.

The pseudo-first-order kinetic (PFO) (Eq. (S1)) and the pseudo-second-order kinetic model (PSO) (Eq. (S2)).

$$\log(q_e - q_t) = \log q_e - \frac{k_1 t}{2.303} \tag{S1}$$

$$\frac{t}{q_t} = \frac{1}{k_2 q_e^2} + \frac{t}{q_e} \tag{S2}$$

where q_e and q_t are the adsorption capacity of CBB at equilibrium and t. The k_1 (min^{-1}) and k_2 (g/(mg min)) are adsorption rate constants of PFO and PSO, respectively.

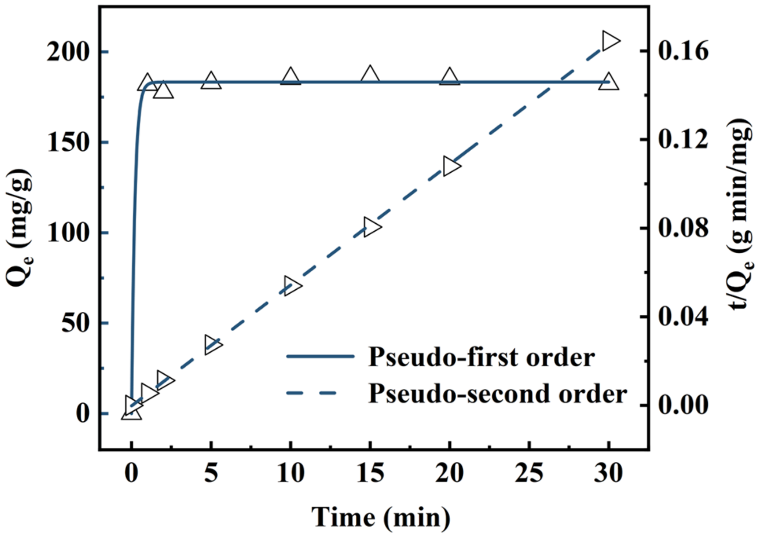

Figure S2. Fitted kinetic curves of Si-MPTS-IL for CBB.

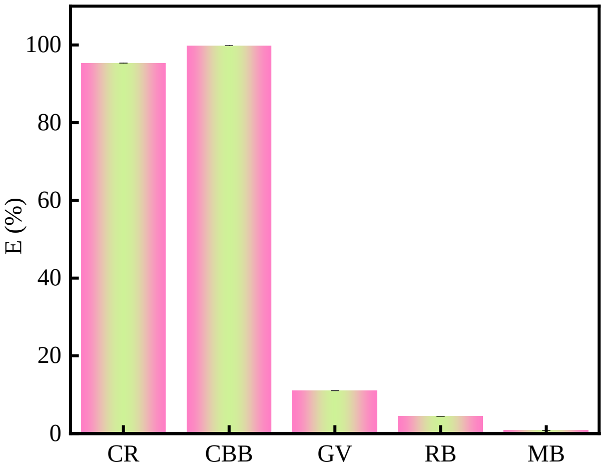

Figure S3. The adsorption performance of Si-MPTS-IL for different dyes.

Table S1. Comparison of adsorption capacity and time of Si-MPTS-IL for CBB and other materials.

Materials	Dye	q_m（mg/g）	t_e（min）	Ref.
TPE-Por-COF	CBB	232.56	50	[1]
TMGO	CBB	249.40	20	[2]
HA microspheres	CBB	83.82	-	[3]
UiO-66-NH_2@Tb-CP	CBB	397.31	-	[4]
PILC-Fe	CBB	9.13	5	[5]
Si-MPTS-IL	CBB	414.44	2	This work

Table S2. Kinetic parameters for CBB adsorption by Si-MPTS-IL.

Dye	Pseudo-first order			Pseudo-second order		
	q_e(mg/g)	k_1(min^{-1})	R^2	q_e(mg/g)	k_2(g/(mg min))	R^2
CBB	183.273	4.822	0.9948	183.486	0.173	0.9998

Table S3. Isotherm fitting parameters of Si-MPTS-IL for CBB.

Dye	Langmuir			Freundlich		
	R^2	q_m (mg/g)	K_L (L/g)	R^2	n	K_F (mg/g)
CBB	0.9960	414.44	0.068	0.7160	4.935	110.845

The Langmuir (Eq. (S3)) and Freundlich (Eq. (S4)) models :

$$C_e / Q_e = C_e / Q_m + 1/(K_L \times Q_m) \tag{S3}$$

$$\ln Q_e = \ln K_F + \frac{1}{n} \ln C_e \tag{S4}$$

where C_e (mg/L) is the equilibrium concentration of CBB (mg/L). Q_e (mg/g) and Q_m (mg/g) denote the equilibrium and theoretical adsorption capacity. The Langmuir and Freundlich constants are expressed by K_L (L/mol) and K_F ((mg/g)/(L/mg)$^{1/n}$), respectively. The Freundlich exponent (n) represents the adsorption intensity.

Table S4. Thermodynamic fitting parameters of adsorption of CBB by Si-MPTS-IL.

Dye	ΔH （KJ/mol）	ΔS (J/(mol · K))	ΔG (KJ/mol)				R^2
			293K	303K	313K	323K	
CBB	16.965	50.715	2.106	1.599	1.091	0.584	0.989

Table S5. Chemical structure and general characterization of CBB.

Dye	Structure	MW (g/mol)	λ_{max} (nm)
CBB	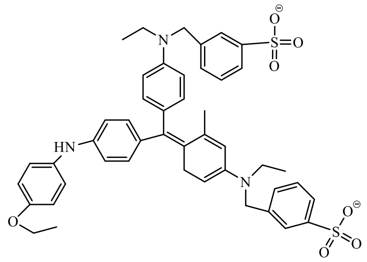	854.04	583

The specific reaction details of the imidazolium ionic liquids modified nano-silica (Si-MPTS-IL) as shown below: The synthesis route of imidazolium ionic liquids modified nano-silica (Si-MPTS-IL) is as follows: 1) Nano-silica particles were prepared using pure TEOS without MPTS by the above method, and we call them F-Si here. Then, 0.3 g of F-Si was weighed and dispersed in 25 mL of anhydrous ethanol. Under magnetic stirring, 50μL ammonia and 400 μL 3-(isobutenyloxy)propyl trimethoxysilane (MPTS) were slowly dripped into the mixture. Subsequently, the mixture was heated to 70 °C for 12 h. The products (named Si-MPTS) were collected by centrifugation. 2) 0.1g of Si-MPTS was weighed into a polyethylene bag for vacuum sealing, and then the 30 wt% [C_2VIm]Br was injected under the condition of nitrogen flow de-oxidation. The electron accelerator (WasiK, USA, Energy 1 MeV, 20 kGy/pass) was used for beam irradiation at 120 kGy. After irradiation, non-reactive monomers and homopolymers were washed for several times with deionized water and anhydrous ethanol. Finally, Si-MPTS-IL was obtained by centrifugation and dried at 50 °C.

References

[1] S. Chang, W. Xie, C. Yao, G. Xu, S. Zhang, Y. Xu, X. Ding, Construction of 2D porphyrin-based covalent organic framework as adsorbent for organic dyes removal and carbon dioxide adsorption, J. Solid State Chem. 304 (2021) 122577. https://doi.org/https://doi.org/10.1016/j.jssc.2021.122577.

[2] M. Sun, J. Ma, M. Zhang, Y. Xiao, Y. Zhu, S. Zhang, Thiourea-modified Fe_3O_4/graphene oxide nanocomposite as an efficient adsorbent for recycling Coomassie brilliant blue from aqueous solutions, Mater. Chem. Phys. 241 (2020) 122450. https://doi.org/https://doi.org/10.1016/j.matchemphys.2019.122450.

[3] H. Wang, H. Xing, K. Yan, D. Han, J. Chen, Oyster shell derived hydroxyapatite microspheres as an effective adsorbent for remediation of Coomassie brilliant blue, Adv.

Powder Technol. 33 (2022) 103425. https://doi.org/https://doi.org/10.1016/j.apt.2022.103425.

[4] Q. Zhang, X. Jiang, A.M. Kirillov, Y. Zhang, M. Hu, W. Liu, L. Yang, R. Fang, W. Liu, Covalent Construction of Sustainable Hybrid UiO-66-NH_2@Tb-CP Material for Selective Removal of Dyes and Detection of Metal Ions, ACS Sustain. Chem. Eng. 7 (2019) 3203–3212. https://doi.org/10.1021/acssuschemeng.8b05146.

[5] A. Kadeche, A. Ramdani, M. Adjdir, A. Guendouzi, S. Taleb, M. Kaid, A. Deratani, Preparation, characterization and application of Fe-pillared bentonite to the removal of Coomassie blue dye from aqueous solutions, Res. Chem. Intermed. 46 (2020) 4985–5008. https://doi.org/10.1007/s11164-020-04236-2.
